# Effect of Alexithymia on Internet Addiction Among College Students: The Mediating Role of Metacognition Beliefs

**DOI:** 10.3389/fpsyg.2021.788458

**Published:** 2022-01-10

**Authors:** Hongge Luo, Yanli Zhao, Jiangyue Hong, Hong Wang, Xiujun Zhang, Shuping Tan

**Affiliations:** ^1^School of Public Health, North China University of Science and Technology, Tangshan, China; ^2^College of Psychology, North China University of Science and Technology, Tangshan, China; ^3^Beijing Huilongguan Hospital, Peking University Huilongguan Clinical Medical School, Beijing, China

**Keywords:** alexithymia, Internet addiction, metacognition, parallel multiple mediator models, college students

## Abstract

**Background:** Previous studies have found that alexithymia plays an important role in the pathogenesis of Internet addiction. However, the effect of alexithymia on both metacognition and Internet addiction has yet to be examined.

**Methods:** The Toronto Alexithymia Scale, Metacognition Questionnaire, and Internet Addiction Test were used to assess a sample of 356 college students. A parallel mediator effect analysis was applied to test the hypothesis that metacognition mediates the relationship between alexithymia and Internet addiction.

**Results:** The parallel multiple mediator models showed that alexithymia predicted the five dimensions of metacognition and Internet addiction, and that three dimensions—cognitive confidence, positive beliefs about worry, and the need to control thoughts—partially mediated this relationship.

**Conclusion:** Alexithymia could directly and indirectly predict Internet addiction *via* metacognition.

## Introduction

The Internet has made work, study, and life, in general, more convenient, but it is also the root of Internet addiction, a serious social issue. Internet addiction refers to the excessive or uncontrolled use of the Internet. It is a pathological form of Internet use that has negative psychological, social, and work effects (Young, 1998). A meta-analysis showed that the prevalence of Internet addiction was 6% globally and 7% in Asian countries (Cheng and Li, 2014). In China, the number of Internet users reached 989 million in December 2020—the largest population being students, which accounts for 21.0% (China Internet Network Information Center, 2020). The prevalence of Internet addiction among Chinese college students was approximately 15.2% (Chi et al., 2016), which is 2.17 times and 2.53 times higher than that of the global and Asian populations, respectively. This shows that college students have a higher incidence and more serious Internet addiction problems. It is now known that excessive use of the Internet seriously affects academic development, physical and mental health, and interpersonal relationships among college students (Ostovar et al., 2016; Cerniglia et al., 2017).

A more thorough investigation of Internet addiction is still lacking. In particular, the factors that influence Internet addiction of college students and the mechanisms between the acting factors and Internet addiction need to be further explored. Examining the issue of Internet addiction by understanding the factors that cause it may be a challenge but doing so could offer evidence-based prevention and the effective control of Internet addiction for college students.

### Alexithymia and Internet Addiction

Previous studies have found that the factors affecting Internet addiction include personality (Kuang et al., 2020), emotion (Yao and Zhong, 2014), and cognition (Weinstein and Lejoyeux, 2010; Şenormanci et al., 2014); among them, personality traits and behavioral patterns play an important role in causing Internet addiction (Rachubińska et al., 2021). Another study proposed that personality factors such as high psychoticism, high harm avoidance, low self-directedness, and low cooperativeness were impact factors for Internet addiction (Weinstein and Lejoyeux, 2010). These personality factors were significantly associated with alexithymia in different populations, such as inpatients who were alcohol-dependent (Evren et al., 2008), psychiatric patients (Grabe et al., 2001), and students (Picardi et al., 2005).

Alexithymia, a set of cognitive features observed in some patients with psychosomatic disorders (Nemiah and Sifneos, 1970; Sifneos, 1973), was first proposed by Nemiah and Sifneos in 1970. It is a unique trait—or more precisely, a complex mixture of traits—that exists in each individual in varying degrees (Vingerhoets et al., 1995; Costa and McCrae, 2013). Different researchers have proposed various descriptions of the structure of alexithymia. Luminet et al. (2018) proposed that the construct includes four components, namely, difficulty identifying feelings (DIF), difficulty describing feelings (DDF), externally orientated thinking (EOT), and difficulty fantasizing/lack of fantasy (DFAN). Later, other researchers also proposed some changes to the concept or measurement structure of alexithymia. Some studies suggested the addition of an extra component, namely, difficulty emotionalizing (DEMO), which measures the degree of emotional arousal induced by emotional events. This view emphasizes that alexithymia includes cognitive and emotional components and identifies its subtypes (Vingerhoets et al., 1995; Vorst and Bermond, 2001). However, Preece et al. (2017) suggested the deletion of the fantasy component and proposed an attentional arousal model of alexithymia, emphasizing the difficulty of individuals to focus on emotional stimuli that cannot identify and evaluate emotions.

Taylor and Bagby (2021) summarized the nature of alexithymia according to a theoretical perspective and empirical research in the latest review, believing that any change should be consistent with the original observations of Nemiah and Sifneos. He proposed that adding the “emotionalizing” component still requires more theoretical and empirical evidence, and the “lack of fantasy” is a more peripheral but essential component of the alexithymia construct.

Regardless, alexithymia might lead to invalid and inflexible emotional regulation modes and become a risk factor for various physical and mental disorders (Luminet et al., 2021), Bonnaire et al. (2013) and Morie et al. (2016) suggested that alexithymia may have a critical role in the pathogenesis of substance use disorders, behavioral addiction, and Internet addiction. Dalbudak et al. (2013), Lyvers et al. (2016), and Schimmenti et al. (2017) found that the level of alexithymia was higher in the Internet addiction group than in the normal group, and that there is a significantly positive correlation between Internet addiction and alexithymia. Mahapatra and Sharma (2018) and Wachs et al. (2020) suggested that alexithymia may be a strong risk factor for Internet addiction. While Dalbudak et al. (2013) and Mahapatra and Sharma (2018) observed the causal relationship between alexithymia and Internet addiction, and the factors that may mediate this relationship remain to be elucidated. Moreover, this relationship has only been studied in Western countries despite the high Internet addiction rates in Asian countries (Cheng and Li, 2014; Mahapatra and Sharma, 2018). Due to cultural differences, Chinese-Canadian students have higher levels of alexithymia than Euro-Canadian students (Dere et al., 2012). Therefore, it is necessary to expand the research on Asian populations and explore cross-cultural differential studies.

### Metacognition and Internet Addiction

Studies have confirmed that metacognition is strongly associated with multiple psychological disorders (Wells, 2013, 2019). Metacognition is the knowledge or belief of the own cognitive system of individuals, the affected functioning of the system, regulation and awareness of current cognitive states, and appraisal of the importance of thought and memory (Flavell, 1979). Wells and Matthews (1996) developed the metacognitive perspective into an understanding of the causes of mental health problems and how they can be treated accordingly. They proposed a metacognitive model of psychological distress—the Self-Regulatory Executive Function model (S-REF) (Wells and Matthews, 1994)—in which a particular cognitive attention syndrome (cognitive attentional syndrome, CAS) causes psychological distress and relapse after treatment. The CAS is a set of poor coping methods, including repetitive negative thinking (worry and rumination), threat monitoring, and associated unbeneficial cognitive and behavioral strategies. It is regulated by positive (arguing that worry, rumination, threat monitoring, and other similar strategies are useful) and negative (“rumination” is uncontrollable) metacognitive beliefs. Wells and Matthews (1994) believed that the self-regulation of CAS regulated by metacognitive beliefs is problematic, and metacognitive beliefs may be a core underlying mechanism of psychological distress.

The metacognitive theory provides a new perspective on psychological issues and facilitates the study of psychopathology as it focuses more on the regulatory role of metacognitive beliefs (Wells and Sembi, 2004; Wells, 2009). Spada et al. (2015) proposed a triphasic metacognitive formulation of the S-REF model to analyze the psychopathological significance of metacognitive beliefs on addictive behaviors. The formulation states that metacognition has an important role in coping styles, leading to the persistence of negative thoughts. Moreover, it plays a central role in the development and maintenance of addictive behavior. The model proposes various aspects of the CAS, such as attentional bias, extended thinking (desire thinking, rumination, and worry), thought suppression, and disruptions in metacognitive monitoring.

There is a significant positive correlation between metacognitive beliefs and different addictive behaviors, such as dependence severity, nicotine use (Spada et al., 2007; Nikčević et al., 2017), and alcohol use (Spada and Wells, 2010). A recent study has also found that metacognitions are associated with the use of problematic social networking sites among young adults (Balıkçı et al., 2020) and late adolescents (Ünal-Aydın et al., 2021). Regarding the relationship between metacognitive beliefs and Internet addiction, one study found that the two positive metacognitions (i.e., escapism and controllability) could mediate the relationship between emotional dysregulation and Internet use; however, this study uses a subscale to measure metacognitive beliefs (Casale et al., 2016). Another study analyzed the full mediating role of metacognition in negative emotion and problematic Internet use, but it did not analyze in depth the role played by different metacognitive beliefs (Spada et al., 2008). Positive and negative metacognitive beliefs play different roles in addiction (Hamonniere and Varescon, 2018); one study further pointed out that specific positive metacognitions and negative metacognitive beliefs were the only significant predictors of Internet addiction, rather than weekly online gaming hours, anxiety, and depression (Spada and Caselli, 2017). Many studies are needed for the relationship between different general metacognitive beliefs and addictive behaviors (Spada et al., 2015). Therefore, it is necessary to adopt a better measurement tool to analyze in depth the relationship of different general metacognitive beliefs with Internet addiction.

### Alexithymia and Metacognition

Alexithymia is a relatively stable and negative personality trait that manifests as low emotional awareness and operational thinking. These are more likely to produce rigid and inflexibility thinking patterns (Taylor and Bagby, 2021) in the individual, make him/her more prone to suppressive regulation strategies rather than reappraisal strategies (Swart et al., 2009), and trigger poor internal monitoring and avoidance behavior (Luminet et al., 2021). Due to different types of metacognitive processing mentioned in the S-REF model, such as the aforementioned characteristics, individuals have two very different cognitive processing modes, namely, object mode and metacognitive mode. In the object mode, the metacognitive belief of the individual is: “My cognitive process is a real and accurate representation of the objective reality, which represents the objective real existence without evaluation.” In the metacognitive mode, the metacognitive belief is: “My cognition of the outside world does not represent the real world; there may be errors, so I need to evaluate my own cognitive process.” The object mode represents a more rigid model, while the metacognitive mode is more flexible. Individuals with psychological stress frequently adopt the object mode (Wells, 2000).

The S-REF model equally applies to individuals with addictive behaviors who are also very likely to adopt the object mode (Spada et al., 2015). Further studies found that alexithymia was significantly associated with different dimensions of metacognition (e.g., risk uncontrollability, cognitive confidence, and need to control thoughts) in different populations, such as high school students (Babaei et al., 2015), and those suffering from nomophobia (Yavuz et al., 2019). However, to date, no studies have investigated the effect of alexithymia on both metacognition and Internet addiction.

This study examined the relationships between alexithymia, metacognition, and Internet addiction. More specifically, we hypothesized that alexithymia could directly or indirectly affect Internet addiction *via* metacognition. In sum, two hypotheses were formulated as follows:

*H1*: Alexithymia statistically predicts Internet addiction.
*H2*: Metacognition plays a mediating role between alexithymia and Internet addiction.

## Materials and Methods

### Participants and Procedure

The survey was conducted with college students from North China University of Science and Technology between February and April 2020. The target population was selected using cluster sampling (stratified by grade and class as the sampling unit). Using electronic structured questionnaires, we assessed sociodemographic variables including sex, age, and grade, as well as aspects of alexithymia, metacognition, and Internet addiction. The questionnaires were anonymous to ensure data reliability and confidentiality. Participants took approximately 15 min to complete the questionnaires.

A total of 400 participants, aged 18–26 years (*M* = 21.19, *SD* = 1.57), filled the survey, and 356 (89% of the initial sample) surveys were valid. Of the participants, 190 were men (53.37%), 85 were senior freshmen (23.88%), 78 were sophomores (21.91%), 93 were juniors (26.12%), and 100 were seniors (28.09%).

### Ethics

Ethical approval for this study was obtained from the Research Ethics Committee of the North China University of Science and Technology. This study was conducted based on the principles of the Declaration of Helsinki.

### Measures

#### Young’s Internet Addiction Test

The Chinese version of Young’s Internet Addiction Test (IAT) is a widely used self-report test that assesses the degree of network addiction. It includes 20 items, each one rated using a 5-point Likert scale (from 1 = never to 5 = always). Higher IAT scores indicate higher levels of problematic Internet use. A total score of 80–100 points indicated an Internet addiction problem, 50–79 points indicated a possible Internet addiction problem, and <30 points indicated no obvious problem. Cronbach’s α for the Chinese version of the IAT was 0.91. An association analysis was performed with the total score of the Chinese Internet Addiction Scale (Chen et al., 2003), with a criterion validity of 0.71. The scale has good psychometric properties (Cao et al., 2010).

#### Toronto Alexithymia Scale (TAS-26)

The Chinese version of the Toronto Alexithymia Scale (TAS) is a self-assessment questionnaire with 26 items used to assess alexithymia, including the following four subscales: F1, difficulty in identifying and distinguishing between feelings and bodily sensations; F2, difficulty in describing feelings; F3, reduced daydreaming; and F4, externally oriented thinking. Each item was evaluated based on the severity of the symptoms (from 1 = completely disagree to 5 = completely agree). Higher TAS scores indicated more severe alexithymia. The test-retest reliability for the Chinese version of the TAS was 0.81–0.84, and it has good psychometric properties (Yao et al., 1992), similar to that reported in another study (Kupfer et al., 2000).

#### Metacognition Questionnaire

Metacognition Questionnaire (MCQ-30) is used to assess generic metacognitive beliefs associated with psychopathology (Wells and Cartwright-Hatton, 2004). The Chinese version of the MCQ-30 was used in this study. It has 30 items, including the following five subscales: Cognitive Confidence (CC), Positive Beliefs about Worry (POS), Cognitive Self-Consciousness (CSC), Negative Beliefs about Uncontrollability and Danger of Worry (NEG), and Need to Control Thoughts (NC). Each item was scored using a 4-point scale (1 = disagree, 2 = somewhat agree, 3 = agree, and 4 = completely agree). Cronbach’s α coefficient, test-retest reliability, and the split-half reliability of the total questionnaire were 0.735–0.897, 0.593–0.741, and 0.715–0.871, respectively. These subscales have acceptable reliability and validity (Fan et al., 2017).

### Statistical Analysis

Data were analyzed using IBM SPSS Statistics (version 23.0; IBM, Armonk, NY, United States). Pearson’s correlations were used to analyze the association between IAT, TAS-26, and MCQ-30. Gender differences in IAT, MCQ with its dimensions, and TAS with its dimensions were analyzed using a *t*-test for continuous variables and a chi-square test for categorical variables.

**FIGURE 1:**
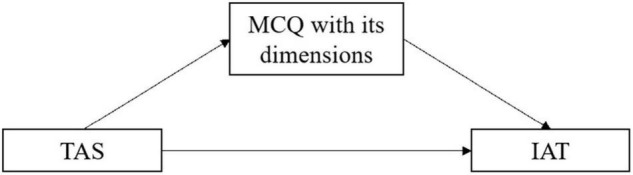
Measurement model.

A parallel mediator effect analysis was performed using Model 4 in the PROCESS macro using SPSS software. Specifically, we analyzed the direct and indirect effects of alexithymia on Internet addiction through the following five dimensions of metacognition: CC, POS, CSC, NEG, and NC. The measurement model is shown in Figure 1. In the parallel mediator effect analysis, alexithymia of participants was evaluated using only the total score. For Model 4, a bootstrapping procedure was selected with 5,000 bootstrap samples. The 95% bootstrap CI (95% CIs) contained zero, indicating that there was no significant effect. The 95% CIs did not contain zero, indicating that there was a significant effect.

Most of the data in this study were obtained from self-reported questionnaires from the same time and place, which may produce common method deviations. Therefore, a common method test for deviation is required before conducting a formal analysis of the collected data. The unrotated exploratory factor analysis extracted 16 factors with a feature root and maximum factor variance interpretation rate of 24.23% (less than 40%); therefore, there is no serious common method deviation in this study.

## Results

### Descriptive Statistics and Partial Correlation Analysis

The descriptive statistics in IAT, MCQ with its dimensions, and TAS with its dimensions are presented in Table 1. Notably, 12% of the participants scored between 80 and 100 on the IAT, and 56% scored between 50 and 79, suggesting that a majority of the participants had moderate to severe Internet addiction problems.

**TABLE 1:** Descriptive statistics and partial correlations between alexithymia, Internet addiction, and metacognition with its dimensions.

Measures	Overall *M* ± *SD or N(%)*	1	2	3	4	5	6	7	8	9	10	11	*12*
1. CC	14.22 ± 3.30	1											
2. POS	14.17 ± 3.30	−0.23***	1										
3. CSC	12.15 ± 2.45	–0.10	0.27***	1									
4. NEG	13.96 ± 3.36	−0.45***	0.29***	0.20***	1								
5. NC	14.65 ± 3.54	−0.41***	0.30***	0.27***	0.64***	1							
6. MCQ	69.15 ± 8.18	–0.08	0.64***	0.57***	0.68***	0.74***	1						
7. F1	23.37 ± 4.63	0.54***	−0.22***	−0.14**	−0.53***	−0.48***	−0.34***	1					
8. F2	18.62 ± 4.47	0.53***	−0.30***	−0.23***	−0.59***	−0.54***	−0.45***	0.76***	1				
9. F3	17.41 ± 2.68	0.27***	−0.21***	−0.26***	−0.30***	−0.36***	−0.34***	0.32***	0.45***	1			
10. F4	28.35 ± 3.30	0.27***	−0.17**	−0.39***	−0.24***	−0.30***	−0.30***	0.30***	0.45***	0.46***	1		
11. TAS	87.75 ± 11.89	0.55***	−0.29***	−0.31***	−0.57***	−0.56***	−0.47***	0.83***	0.90***	0.65***	0.67***	1	
12. IAT	59.18 ± 16.86	0.45***	−0.31***	−0.23***	−0.49***	−0.54***	−0.45***	0.46***	0.51***	0.43***	0.32***	0.56***	1
scores of IAT: <50		113 (32%)											
scores of IAT: 50 ∼ 79		199 (56%)											
scores of IAT: 80∼100		44 (12%)											

*CC, cognitive confidence; POS, positive beliefs about worry; CSC, cognitive self-consciousness; NEG, negative beliefs about uncontrollability and danger of worry; NC, need to control thoughts; MCQ, metacognition questionnaire; F1, difficulty in identifying and distinguishing between feelings and bodily sensations; F2, DDF; F3, reduced daydreaming; F4, externally oriented thinking; TAS, Toronto Alexithymia Scale; IAT, Internet addiction test.*

**p < 0.01, ***p < 0.001.

Pearson’s *r* correlations between alexithymia, Internet addiction, and metacognition and its dimensions are presented in Table 1. After controlling for gender and grade, the IAT score was significantly and positively related to both cognitive confidence and the TAS scores. Moreover, the IAT scores were significantly and negatively related to both the scores of the remaining subscales of the MCQ and the total scores of the MCQ. Notably, the TAS score was significantly associated with the MCQ scores and its subscales.

### Analysis of Mediator Model

We analyzed the indirect effect of the dimensions of metacognition on the relationship between alexithymia and Internet addiction because there were significant correlations between them. The regression analysis results can be found in Table 2.

**TABLE 2:** Regression analysis of variables in the model.

Equation		Fit index			95%*CI*			
Outcome variable	Predictive variable	*R* ^2^	*F*	β	Lower limit	Upper limit	*t*	*p*
IAT	TAS	0.31	161.08	0.56	0.67	0.92	12.69	< 0.000
TAS	CC	0.31	155.64	0.55	0.13	0.18	12.48	< 0.000
	POS	0.09	34.77	–0.30	–0.11	–0.06	–5.90	< 0.000
	CSC	0.10	40.54	–0.32	–0.09	–0.05	–6.37	< 0.000
	NEG	0.32	166.20	–0.57	–0.18	–0.14	–12.89	< 0.000
	NC	0.31	160.31	–0.56	–0.19	–0.14	–12.66	< 0.000
TAS				0.26	0.21	0.53	4.50	< 0.000
CC				0.14	0.23	1.25	2.86	< 0.000
POS				–0.10	–0.93	–0.04	–2.13	0.03
CSC	IAT	0.42	41.56	–0.02	–0.76	0.44	–0.52	0.60
NEG				–0.08	–0.96	0.17	–1.38	0.17
NC				–0.25	–1.71	–0.64	–4.32	< 0.000

*CC, cognitive confidence; POS, positive beliefs about worry; CSC, cognitive self-consciousness; NEG, negative beliefs about uncontrollability and danger of worry; NC, need to control thoughts; TAS, Toronto Alexithymia Scale; IAT, Internet addiction test.*

Alexithymia could positively predict Internet addiction [β = 0.56, 95% CI (0.67, 0.92)]. When alexithymia and the five dimensions of metacognition were included in the regression equation, the predictive effect of alexithymia on Internet addiction was still significant [β = 0.26, 95% CI (0.21, 0.53)]. Alexithymia directly predicted CC [β = 0.55, 95% CI (0.13, 0.18)], POS [β = −0.30, 95% CI (−0.11, −0.06)], CSC [β = −0.32, 95% CI (−0.09, −0.05)], NEG [β = −0.57, 95% CI (−0.18, −0.14)], and NC [β = −0.56, 95% CI (−0.19, −0.14)]. CC [β = 0.14, 95% CI (0.23, 1.25)], POS [β = −0.10, 95% CI (−0.93, −0.04)], and NC [β = −0.25, 95% CI (−1.71, −0.64)] significantly predicted Internet addiction, while CSC [β = −0.02, 95% CI (−0.76, 0.44)] and NEG [β = −0.08, 95% CI (−0.96, 0.17)] did not.

The direct effect of alexithymia on Internet addiction was 0.37. The indirect effect analysis showed that the dimensions of metacognition partially accounted for the relationship between alexithymia and Internet addiction, with a total indirect effect value of 0.42. Specifically, the indirect effect consisted of five indirect paths, of which the three pathways are significant: the indirect effect of CC (Effect = 0.11) from the path of alexithymia → cognitive confidence → Internet addiction, the indirect effect of POS (Effect = 0.04) from the path of alexithymia → positive beliefs about worry → Internet addiction; the indirect effect of NC (Effect = 0.20) from the path of alexithymia → need to control thoughts → Internet addiction. The three significant indirect effects accounted for 14, 8, and 25% of the total effect, respectively. The indirect effect of CSC and that of NEG were not significant. The results of the effect analysis are shown in Table 3.

**TABLE 3:** Total effect, direct effect, and indirect effect analysis of alexithymia and metacognition with its dimensions.

	Effect	Boot SE	Boot LLCI	Boot ULCI	Relative effect (%)
Total effect	0.79	0.06	0.67	0.92	
Direct effect	0.37	0.08	0.21	0.53	0.47
Total indirect effect	0.42	0.06	0.30	0.55	0.53
Indirect effect of CC	0.11	0.04	0.03	0.20	0.14
Indirect effect of POS	0.04	0.02	0.00	0.09	0.05
Indirect effect of CSC	0.01	0.02	–0.03	0.05	0.01
Indirect effect of NEG	0.06	0.05	–0.04	0.17	0.08
Indirect effect of NC	0.20	0.05	0.10	0.29	0.25
Comparison 1	0.05	0.04	–0.03	0.12	
Comparison 2	–0.06	0.05	–0.15	0.03	
Comparison 3	–0.11	0.04	–0.18	–0.03	

*CC, cognitive confidence; POS, positive beliefs about worry; CSC, cognitive self-consciousness; NEG, negative beliefs about uncontrollability and danger of worry; NC, need to control thoughts; Comparison 1, indirect effect of CC minus indirect effect of POS; Comparison 2, indirect effect of CC minus indirect effect of NC; Comparison 3, negative indirect effect of POS minus indirect effect of NC.*

**FIGURE 2:**
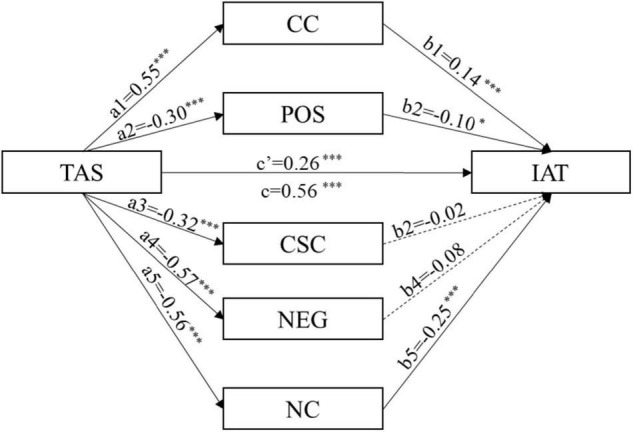
Mediation model of alexithymia, Internet addiction, and metacognition with its dimensions. TAS, Toronto Alexithymia Scale; CC, cognitive confidence; POS, positive beliefs about worry; CSC, cognitive self-consciousness; NEG, negative beliefs about uncontrollability and danger of worry; NC, need to control thoughts; IAT, Internet addiction test; *p < 0.05, ^***^p < 0.001; the continuous lines mean that the path coefficient between the variables is significant. The dotted lines mean that the path coefficient between the variables is not significant.

There was neither significant difference between the indirect effect of CC and that of POS nor between the indirect effect of CC and that of NC. The indirect effect of NC is greater than that of POS. Figure 2 shows the path model.

## Discussion

This study explores the role of alexithymia and metacognition in the pathogenesis of Internet addiction. Both hypotheses were confirmed in this study. It was found that alexithymia can predict Internet addiction directly and indirectly. A parallel mediation analysis showed that the three dimensions of metacognition partially mediated the relationship between alexithymia and Internet addiction. Furthermore, the significant indirect effect included three paths, namely, the indirect effects of CC, POS, and NC.

### Direct Effect

Consistent with *H1*, alexithymia can directly and positively predict Internet addiction. Specifically, the more serious the alexithymia, the higher the degree of Internet addiction, which is consistent with previous results found in studies conducted in Western countries (Dalbudak et al., 2013; Lyvers et al., 2016; Schimmenti et al., 2017). This may be due to the following reasons.

First, alexithymia, as a stable personality trait, may be a predisposition to psychiatric illness (Bagby and Taylor, 2009) and include addictive behaviors (El-Rasheed et al., 2017). It may be influenced by factors such as life experiences, early traumatic experiences, and unsafe attachment (Thorberg et al., 2016), and deficient interoceptive awareness associated with high alexithymia (Brewer et al., 2016) may mean that there is limited recognition of internal cues of overconsumption, which in turn may increase the likelihood and vulnerability of addiction disorders (Lyvers et al., 2019).

Second, individuals who lack an intrinsic emotional experience prefer to focus on and amplify the physiological components of emotional arousal, while their insufficient prefrontal cognitive control capabilities (Orsolini, 2020) are more likely to relieve tension through binge eating, substance abuse, and other compulsive activities (Taylor et al., 1989, 1991). In the context where Internet use is extremely common, these individuals are more likely and more easily attracted to the network, resulting in Internet addiction.

Third, alexithymia may induce in individuals more negative emotions and the poor ability to psychologically reconstruct emotions (Swart et al., 2009), which may become cognitive deficits in processing emotional information (Fantini-Hauwel et al., 2015). They may resort to emotional regulation strategies of expression suppression, which may increase impulsive or compulsive behavior. Excessive Internet use may also be used as a maladaptive coping strategy (Blasi et al., 2019) to regulate both the cognitive and affective states to avoid and relieve stress (de Berardis et al., 2009; Balıkçı et al., 2020). In addition, individuals with alexithymia have difficulty identifying, expressing, and communicating emotions and are unwilling to interact directly with others, leading to their poor social and interpersonal functions (Gross, 2002). They prefer to communicate online to reduce the need for publicly shared emotions, and thus meet their unfulfilled social needs (Samur et al., 2013).

### Indirect Effect

With regard to *H2*, the three dimensions of metacognition partially mediated the relationship between alexithymia and Internet addiction; the mediation is arranged from large to small for the indirect effects of NC, CC, and POS. This is in general agreement with previous studies where the “need to control thoughts” and “lack of cognitive confidence” can effectively predict addictive behavior (Spada et al., 2015). Among them, positive and negative metacognitive beliefs play different roles in Internet addiction (Veeraraghavan, 2009).

Above all, alexithymia is a negative personality trait, and the thinking pattern of individuals with this personality trait is rigid. They adopt regulatory strategies of inhibition rather than revaluation. The object model proposed by Wells is an inflexible and unnecessary revaluation. That is to say, alexithymia may be the source or personality basis of this processing mode where individuals identify certain ideas and negative emotional experiences as reality and tend to use both attention resources and coping strategies to change “reality.” This strengthens their original negative metacognitive beliefs, which will then promote the emergence of addictive behaviors. The following are specific explanations of the three significant paths.

The first path, which is the largest indirect effect, is that alexithymia can predict Internet addiction through the “need to control thoughts,” that is, the more severe the alexithymia, the less the individual controls their thoughts, and the higher the network addiction. Alexithymia results in control deficits. As alexithymia scores increased, the ability to effectively inhibit control declined. Furthermore, the factors of “externally oriented thinking (EOT)” (Dressaire et al., 2015) and “DIF” (Battista et al., 2021; Correro et al., 2021) of alexithymia play a major role in terms of inhibiting control. Individuals who scored high on these two factors lack internal awareness and representation (Rinaldi et al., 2017); they cannot perform internal monitoring (Correro et al., 2021) and exert less internal control.

Metacognitive beliefs, specifically the “need to control thoughts,” act on the three stages of the metacognitive model of Internet addiction according to the triphasic metacognitive formulation of addictive behaviors. The three stages are pre-engagement, engagement, and post-engagement. In the pre-engagement phase, the negative metacognitive belief of the “need to control thoughts” emerges, (e.g., “I cannot control my thoughts of going online”). It will instead activate the idea of inhibiting Internet access (Wenzlaff and Wegner, 2000). If maladaptation also leads to the emergence of negative emotions, individuals who access the Internet are more likely to regulate their emotions and escape the increasing differences between the current suppressed state and desire to access the Internet. During the engagement phase, positive metacognitive beliefs (e.g., “Internet access will help me control my thoughts/reduce my worries”) and poor metacognitive monitoring lead to a decline in individual ability to regulate behaviors. As the severity of addictive behaviors increases the emergence of negative metacognitive beliefs about its uncontrollability (e.g., “Once I start using the Internet, I find it difficult to stop”) leads to its persistence, which contributes to its perseveration. During the post-engagement phase, an invasion (e.g., self-blame thinking or withdrawal symptoms) leads to obtaining positive metacognitive beliefs about post-engagement reflection (e.g., “If I analyze why I feel this way, I will understand why I am using the Internet”) and activates the coping style of thought suppression and rumination. This coping style leads to a deterioration of the negative effects, thus increasing the possibility of Internet access as a means of self-regulation.

A longitudinal study found that lower inhibitory control could predict an increase in Internet gaming time after a year (Kräplin et al., 2021). Therefore, individuals fail to intentionally control their own thoughts and behaviors, including Internet behavior. In addition, individuals with alexithymia may have potential behavioral adaptation barriers; they might not be able to adjust their behavior in a timely manner to adapt to the environment (Zhang et al., 2012). These mechanisms make them more prone to Internet addiction.

The second path is that alexithymia can predict Internet addiction through “cognition confidence.” Specifically, the more severe the alexithymia, the lower the self-evaluation of cognitive ability of individuals, and the higher the Internet addiction. This is consistent with previous results; existing studies have found that alexithymia is correlated with self-confidence in normal respondents and alcoholics (Loas et al., 2000). Individuals with higher alexithymia scores did not differ from those with lower alexithymia scores in their tasks but were significantly less confident about their decisions (Lorey et al., 2012). Individuals with alexithymia cannot accurately identify and describe their emotional states. This increases uncertainty and their tendency to be isolated (Lorey et al., 2012), so they often underestimate their abilities.

Self-efficacy refers to the speculation and judgment of the ability of an individual to accomplish a certain behavior. According to the formulation of addictive behaviors, having low self-efficacy or metacognitive confidence was found to increase the risk of Internet addiction (Kuss et al., 2014; Chen et al., 2020). Individuals with low self-efficacy and metacognitive confidence may use the network as a tool to search for specific information. They can achieve a virtual and temporary sense of accomplishment by spending a lot of time online and improving self-effectiveness or reducing metacognitive discomfort (Muris, 2002; Iskender and Akin, 2010; Spada et al., 2015).

The third path is that alexithymia can predict Internet addiction through “positive beliefs about worry.” As a positive belief in metacognition, it plays a significant, but small mediating role. Furthermore, the more severe the alexithymia, the more positive the beliefs about worry, and the higher the network addiction; having positive beliefs about worry means valuing thoughts with worry content. Previous studies have found high levels of trait anxiety and state anxiety in individuals with high alexithymia (Franzoi et al., 2020). The same result was revealed for mobile phone addiction (Gao et al., 2018). In addition, difficulties in identifying feelings can predict the level of anxiety later in life (Oakley et al., 2020). We further discovered through the metacognitive scale that people with high alexithymia may not only focus on the mood of anxiety but also on its benefits. They think that worry can lead to good results and prevent danger. According to the formulation of addictive behaviors, positive metacognitive beliefs, such as thinking that worrying can bring good results and prevent danger, always work in the pre-engagement phase; thus, individuals resort to the coping mechanism of rumination, thought suppression, and threat monitoring. They think about the reason, consequence, and meaning behind their Internet use or try to control their urge to go online. However, this instead increases the level of worry and desire to go online. Therefore, individuals will enter the engagement phase by alleviating their worries through the Internet. In the post-engagement phase, they will have developed more serious anxiety about their online behaviors. More severe rumination and thought suppression further promote their access to the Internet, and a vicious cycle is eventually formed (Spada et al., 2015).

### Limitations and Future Perspectives

There are some limitations and future perspectives in this study. First, alexithymia plays an important role in the occurrence of Internet addiction, so it seems important to improve their ability to identify and describe oneself and emotions of others in preventing and interfering in Internet addiction. Additionally, alexithymia is a relatively stable personality structure that is not easy to change. Individuals with alexithymia have poor psychotherapy responses due to their poor understanding of mood changes (Parker et al., 1989), thus it is difficult to prevent Internet addiction by improving alexithymia. Wells et al. (2020) proposed the use of metacognitive therapy based on S-REF models, which can effectively treat multiple psychological disorders by improving dysfunctional metacognition beliefs. Therefore, future studies can take interventions to prevent Internet addiction through metacognitive therapy.

Second, several studies have proposed a potential universal contribution of metacognition to persistent and unhealthy forms of cognition and behavior (Spada et al., 2007; Casale et al., 2016). The MCQ-30 only measures general metacognitive beliefs and is not intended to specifically capture metacognitions in any addiction behaviors. However, for online games, for example, a metacognitive belief scale has already been developed (Spada and Caselli, 2017). Moreover, only the total score of TAS was used in the parallel mediation model. In addition, this study follows a cross-sectional design and currently only makes preliminary inferences based on the statistical results. This is because not all participants go through the same process of Internet addiction since only some have problems with Internet use. To investigate more deeply the psychopathology mechanisms of Internet addiction, a longitudinal tracking design is needed.

Third, there are different types of Internet addiction, such as game addiction, shopping addiction, gambling, and social tools. In this study, higher scores of IAT and TAS do not rule out that individuals are only repeating these previous bad habits, so future studies could attempt to identify whether different metacognitive beliefs can have an impact on various types of Internet addiction. In addition, questions about the content of the Internet could also be further explored.

Fourth, the general mood measures were not assessed, especially during the pandemic, which may also be a key predictor of addiction. Future studies should control for or include these variables in order to obtain more comprehensive results.

Last but not least, only 356 college students were evaluated by way of collecting the data online. The sample size and the population were relatively small; thus, future research with a larger sample is needed to improve reliability. In addition, due to cultural differences, alexithymia may measure different contents between Eastern and Western cultures, so future research should consider the impact of cultural differences on the results.

## Conclusion

This study investigated the influence of alexithymia on Internet addiction and its pathogenesis of underlying psychological factors. The following conclusion were drawn: (1) alexithymia could directly predict Internet addiction and metacognition, and (2) metacognitions partially mediate the relationship between alexithymia and Internet addiction, including three paths, the indirect effects of CC, POS, and NC, which extends conceptualizations of the problem that are based on a direct link between alexithymia and Internet addiction.

## Data Availability Statement

The original contributions presented in the study are included in the article/supplementary material, further inquiries can be directed to the corresponding authors.

## Ethics Statement

The studies involving human participants were reviewed and approved by the Research Ethics Committee of the North China University of Science and Technology. The patients/participants provided their written informed consent to participate in this study.

## Author Contributions

HL, XZ, and ST developed the study concept. HW, YZ, and JH performed testing and data collection. HL analyzed the data and drafted the manuscript. All authors contributed to the article and approved the submitted version.

## Conflict of Interest

The authors declare that the research was conducted in the absence of any commercial or financial relationships that could be construed as a potential conflict of interest.

## Publisher’s Note

All claims expressed in this article are solely those of the authors and do not necessarily represent those of their affiliated organizations, or those of the publisher, the editors and the reviewers. Any product that may be evaluated in this article, or claim that may be made by its manufacturer, is not guaranteed or endorsed by the publisher.
